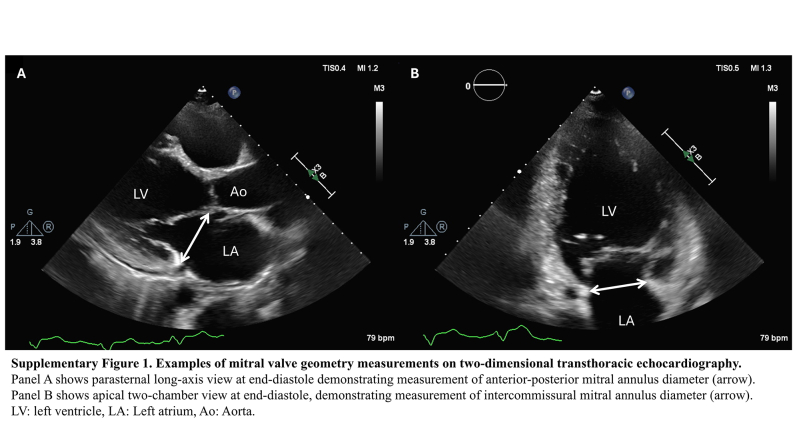# Impact of Baseline Anteroposterior Mitral Annular Dimensions on Clinical Outcomes after MitraClip for Secondary Mitral Regurgitation

**DOI:** 10.1016/j.shj.2025.100460

**Published:** 2025-03-20

**Authors:** Jason H. Rogers, Thomas W. Smith, Jeroen J. Bax, Federico M. Asch, D. Scott Lim, Ningyan Wong, Janani Aiyer, William T. Abraham, JoAnn Lindenfeld, Michael J. Mack, Gregg W. Stone, Steven F. Bolling

**Affiliations:** a Division of Cardiovascular Medicine, University of California, Davis Medical Center Sacramento, California, USA; b Department of Cardiology, Leiden University Medical Center, Leiden, the Netherlands; c Cardiovascular Core Laboratories and Cardiac Imaging Research, MedStar Health Research Institute, Washington, District of Columbia, USA; d Division of Cardiovascular Medicine, University of Virginia, Charlottesville, Virginia, USA; e Advanced Cardiac Valve Center, University of Virginia, Charlottesville, Virginia, USA; f Abbott Structural Heart, Santa Clara, California, USA; g Division of Cardiovascular Medicine, Ohio State University Medical Center, Columbus, Ohio, USA; h Division of Cardiology, Vanderbilt University Medical Center, Nashville, Tennessee, USA; i Division of Cardiothoracic Surgery, Baylor Scott & White Research Institute, Plano, Texas, USA; j Zena and Michael A. Wiener Cardiovascular Institute, Icahn School of Medicine at Mount Sinai, New York, New York, USA; k Department of Cardiac Surgery, University of Michigan, Ann Arbor, Michigan, USA

**Keywords:** Mitral annulus, Mitral regurgitation, Mitral valve repair, Transcatheter edge-to-edge repair (TEER)

## Abstract

**Background:**

In the randomized Cardiovascular Outcomes Assessment of the MitraClip Percutaneous Therapy for Heart Failure Patients with Functional Mitral Regurgitation (COAPT; NCT01626079) trial, mitral transcatheter edge-to-edge repair (M-TEER) improved clinical outcomes in patients with severe secondary mitral regurgitation (MR). A prior post hoc analysis from the COAPT trial showed that increasing anteroposterior mitral annular diameter (APMAD) was the sole independent echocardiographic predictor of the composite endpoint of death or heart failure hospitalizations (HFH) at 2 years. Given the relationship between the mitral annulus and leaflets, we examined the association of baseline APMAD with long-term clinical outcomes.

**Methods:**

COAPT patients (n = 575) were stratified into tertiles by baseline APMAD as follows: small APMAD, medium APMAD, and large APMAD. APMAD was measured in the anteroposterior direction from the parasternal long-axis view at end-diastole and in the intercommissural direction from the apical two-chamber view.

**Results:**

Patients with larger baseline APMAD were more often male and had fewer comorbidities, larger ventricles, and larger mitral orifice areas. At 2 years, there were no significant differences in MR severity and the composite endpoint of death or HFH in patients treated with M-TEER by baseline APMAD. In patients treated with guideline-directed medical therapy (GDMT) alone, there were no significant differences in MR severity, but the composite endpoint of death or HFH was higher in patients with the largest baseline APMADs. The treatment effect of M-TEER was consistent among APMAD tertiles (*p*_interaction_ = 0.87).

**Conclusions:**

APMAD was a predictor of adverse outcomes in patients treated with GDMT alone. M-TEER reduced MR severity and the risk of death or HFH regardless of baseline APMAD compared with GDMT alone.

## Highlights

• Mitral transcatheter edge-to-edge repair (M-TEER) is effective across a range of anteroposterior mitral annular diameters (APMADs).
• Mitral regurgitation and death/HFH are reduced by M-TEER regardless of APMAD.
• Functional capacity and quality of life are also significantly improved at 2 years.

## Introduction

Patients with secondary mitral regurgitation (SMR) experience a poor prognosis, reduced quality of life, frequent heart failure hospitalizations (HFHs), and increased mortality.^1^ The pathophysiology of SMR is characterized by complex anatomic changes, including impaired left ventricular (LV) systolic function, LV cavity enlargement, papillary muscle displacement with leaflet tethering, and mitral annular dilation.^2^, ^3^, ^4^ In SMR, annular enlargement occurs in the anterior–posterior dimension; this geometric alteration is a primary cause for reduced leaflet coaptation.^5^^,^^6^

Mitral annuloplasty is routinely performed during surgical mitral valve repair to reduce annular dimensions and restore mitral leaflet coaptation.^7^, ^8^, ^9^, ^10^, ^11^ Transcatheter annuloplasty technologies have also been studied as alternatives for treating anterior–posterior dilatation.^12^, ^13^, ^14^ Mitral transcatheter edge-to-edge repair (M-TEER) with the first-generation MitraClip device has been shown to be safe and effective in improving clinical outcomes in HF patients with symptomatic severe SMR despite the use of maximally tolerated guideline-directed medical therapy (GDMT) in the Cardiovascular Outcomes Assessment of the MitraClip Percutaneous Therapy for Heart Failure Patients with Functional Mitral Regurgitation (COAPT) trial.^15^^,^^16^ Furthermore, numerous studies have highlighted the beneficial effects of M-TEER on LV and mitral valve geometry, particularly with new device generations.^17^, ^18^, ^19^

A previously published post hoc analysis of 29 baseline mitral valve echocardiographic parameters from the COAPT trial found that a larger baseline anteroposterior mitral annular diameter (APMAD) was the sole independent predictor of the composite all-cause mortality or HFH and HFH alone.^20^ However, the mechanistic influence of baseline mitral annular diameter on outcomes is not well understood. The present analysis evaluates the effect of baseline APMAD on clinical outcomes among patients in the COAPT trial with severe, symptomatic SMR who were treated with the first-generation MitraClip device.

## Materials and Methods

### Study Design and Study Population

The COAPT randomized controlled trial was a multicenter trial that assessed the therapeutic effect of M-TEER using the first-generation MitraClip system (Abbott, Santa Clara, California) in symptomatic HF patients with moderate-to-severe (3+) or severe (4+) SMR despite maximally tolerated GDMT. The participating centers, investigators, and full study details have been previously published.^15^ The study is registered in Clinicaltrials.gov (NCT01626079) and complies with the latest good clinical practice standards of the Declaration of Helsinki. All patients provided written informed consent. The study was approved by the investigational review boards of the participating countries depending on the national requirements.

A total of 614 patients were randomized in a 1:1 ratio to either M-TEER with the MitraClip device plus GDMT (n = 302) or GDMT alone (n = 312) and were followed for 5 years.^16^ The present analysis focuses on outcomes through 2 years before crossovers were allowed per protocol. For this study, patients enrolled in the COAPT trial were stratified into tertiles (3 equal groups) in increasing order of their baseline APMAD. Patients in tertile 1 were characterized by small APMADs (S-MAD), tertile 2 by medium APMADs (M-MAD), and tertile 3 by large APMADs (L-MAD).

### Echocardiography

Echocardiographic images were evaluated by an independent core laboratory (MedStar Health Research Institute, Washington, DC) for assessment of MR severity and ventricular structure and function per the American Society of Echocardiography guidelines.^21^ For the current analysis, additional core laboratory echocardiographic analyses were performed on all baseline transthoracic echocardiograms, as described previously.^20^ The diameters of the mitral valve annulus were measured from the hinge points in the anteroposterior direction from the parasternal long-axis view and in the intercommissural direction from the apical 2-chamber view at the end-diastole (Supplementary Figure 1). The analysis was performed using commercial software (TomTec-Arena, version 20.18, 2017). To ensure the reproducibility of mitral valve echocardiographic measurements, a randomly selected sample of 20 cases was studied, and intraobserver and interobserver agreement was calculated, as reported in a previous publication.^20^

### Clinical Outcomes

The key outcome of the present analysis was the composite of all-cause death or HFH at a 2-year follow-up. Other clinical outcomes included all-cause mortality, first HFH, cardiovascular death/HFH, functional class assessed using the New York Heart Association (NYHA) functional classification, and quality of life assessed by the Kansas City Cardiomyopathy Questionnaire Overall Summary (KCCQ-OS) score. Adverse events were adjudicated by an independent clinical events committee.

### Statistical Analysis

Continuous data were summarized as mean ​± ​SD or median (25th percentile and 75th percentile). Analysis of variance was used to compare continuous variables between patients in the three groups. Chi-square or Fisher exact test was used to compare categorical variables, which were expressed as percentages and frequencies. Event rates were estimated by Kaplan–Meier analysis and compared with log-rank tests. Cox proportional hazard regression models were used to calculate hazard ratios and 95% CIs. Interaction testing was performed to examine differences in the relative treatment effect of M-TEER vs. GDMT in the three groups. All data were analyzed in the intention-to-treat population. All *p* values are two-sided, and a *p* value <0.05 was considered statistically significant. Statistical analyses were performed using SAS software, version 9.4 (SAS Institute, Cary, North Carolina).

## Results

### Analysis Population and Baseline Characteristics

Of 575 patients with evaluable APMAD at baseline, there were 191 patients in the S-MAD group (M-TEER: 99, GDMT alone: 92), 194 patients in the M-MAD group (M-TEER: 85, GDMT alone: 109), and 190 patients in the L-MAD group (M-TEER: 100, GDMT alone: 90). The mean APMAD in these 3 groups was 2.9 ​± ​0.2 cm in S-MAD, 3.2 ​± ​0.1 cm in M-MAD, and 3.7 ​± ​0.3 cm in the L-MAD group; APMAD by median (IQR) for each group was 2.90 (2.78, 3.00), 3.21 (3.14, 3.31), and 3.60 (3.50, 3.79), respectively. Thirty-nine patients did not have evaluable APMAD due to poor echo quality.

The baseline characteristics for the three groups are presented in Table 1. Patients with larger APMAD were more often male, with significantly larger effective regurgitant orifice areas and LV volumes. Diabetes and renal disease were less frequent in patients with larger APMADs, but prior atrial fibrillation was more common. There were no significant differences in quality of life (KCCQ score and 6-minute walk distance) and functional class (NYHA) between the three groups at baseline.

**Table 1:** Baseline characteristics

	S-MAD (N = 191)	M-MAD (N = 194)	L-MAD (N = 190)	*p* value
Age (years)	70.9 ​± ​11.6	73.3 ​± ​10.7	71.8 ​± ​11.5	0.13
Sex, female	48.2% (92)	34.5% (67)	25.8% (49)	<0.0001
Body mass index (kg/m^2^)	26.4 ​± ​5.7	27.0 ​± ​6.0	27.5 ​± ​6.1	0.24
Body surface area (m^2^)	1.8 ​± ​0.3	1.9 ​± ​0.2	2.0 ​± ​0.2	<0.0001
STS repair score (%)	5.8 ​± ​5.9	6.0 ​± ​5.3	5.4 ​± ​5.4	0.60
STS replacement score (%)	8.3 ​± ​5.6	8.4 ​± ​6.0	7.7 ​± ​6.0	0.43
BNP (pg/mL)	993.9 ​± ​1108.3	1090.5 ​± ​1408.2	1023.4 ​± ​930.6	0.78
Diabetes	44.0% (84)	38.1% (74)	29.5% (56)	0.01
Renal disease	65.4% (125)	56.7% (110)	47.9% (91)	0.003
Coronary artery disease	70.7% (135)	73.7% (143)	70.5% (134)	0.74
Hypertension	77.5% (148)	83.0% (161)	79.5% (151)	0.39
History of atrial fibrillation	41.9% (80)	55.7% (108)	61.6% (117)	0.0004
Prior HFH within 12 months	60.7% (116)	57.2% (111)	51.1% (97)	0.16
EROA (cm^2^)	0.39 ​± ​0.15	0.39 ​± ​0.13	0.43 ​± ​0.17	0.01
LVESV (mL)	122.5 ​± ​51.5	137.6 ​± ​58.9	147.5 ​± ​61.9	0.0002
LVEDV (mL)	176.1 ​± ​65.5	194.6 ​± ​72.4	210.3 ​± ​72.6	<0.0001
LVESVi (mL/m^2^)	66.4 ​± ​25.4	72.3 ​± ​30.2	76.2 ​± ​30.9	0.006
LVEDVi (mL/m^2^)	95.5 ​± ​31.3	102.1 ​± ​35.8	108.0 ​± ​35.2	0.003
LVESD (cm)	5.1 ​± ​0.8	5.4 ​± ​0.9	5.4 ​± ​0.9	0.002
LVEDD (cm)	6.0 ​± ​0.7	6.2 ​± ​0.7	6.4 ​± ​0.7	<0.0001
LVESDi (cm/m^2^)	2.8 ​± ​0.5	2.9 ​± ​0.5	2.8 ​± ​0.5	0.52
LVEDDi (cm/m^2^)	3.3 ​± ​0.4	3.3 ​± ​0.5	3.3 ​± ​0.5	0.88
APMAD (cm)	2.88 ​± ​0.15	3.22 ​± ​0.10	3.69 ​± ​0.29	<0.0001
6MWD (m)	237.4 ​± ​125.2	239.9 ​± ​127.1	251.3 ​± ​123.1	0.52
NYHA class III or IV	63.2% (120)	59.3% (115)	58.9% (112)	0.65
KCCQ-OS score	51.0 ​± ​23.5	52.5 ​± ​23.3	53.6 ​± ​22.0	0.54

*Note.* Data are shown as mean +/− SD or % (n); significance was tested by chi-square or Fisher exact test (categorical variables, as appropriate) or ANOVA (continuous variables).

Abbreviations: 6MWD, six-minute walk distance; ANOVA, analysis of variance; APMAD, anteroposterior mitral annular diameter; BNP, B-type natriuretic peptide; EROA, effective regurgitant orifice area; HFH, heart failure hospitalization; KCCQ-OS, Kansas City Cardiomyopathy Questionnaire Overall Summary; L-MAD, large mitral annular diameter; LVEDD, left ventricular end-diastolic dimension; LVEDDi, left ventricular end-diastolic dimension index; LVEDV, left ventricular end-diastolic volume; LVEDVi, left ventricular end-diastolic volume index; LVESD, left ventricular end-systolic dimension; LVESDi, left ventricular end-systolic dimension index; LVESV, left ventricular end-systolic volume; LVESVi, left ventricular end-systolic volume index; M-MAD, medium mitral annular diameter; NYHA, New York Heart Association; S-MAD, small mitral annular diameter; STS, Society of Thoracic Surgeons.

### Procedural and Safety Outcomes

As shown in Tables 2 and 3, patients in the M-MAD group had a significantly higher number of clips implanted than patients in the S-MAD group (P_S-MAD_ vs. _M-MAD_ = 0.007), although differences among S-MAD vs. L-MAD and M-MAD and L-MAD were not significant (P_S-MAD_ vs._L-MAD_ = 0.21 and P_M-MAD_ vs._L-MAD_ = 0.20). There were no significant differences in the procedure and device times. Regardless of APMAD tertile, there were no new device-specific complications between 30 days and 2 years and there was a low rate of progressive HF-related complications through 30 days and 2 years.

**Table 2:** M-TEER outcomes by baseline APMAD

Procedural outcomes in M-TEER patients	S-MAD (N = 99)	M-MAD (N = 85)	L-MAD (N = 100)	*p* value
Number of MitraClip devices implanted	1.6 ​± ​0.6	1.8 ​± ​0.6	1.7 ​± ​0.7	0.04
Procedure time (min)	168.6 ​± ​162.3	155.2 ​± ​64.6	170.3 ​± ​86.2	0.63
Device time (min)	84.2 ​± ​115.3	77.2 ​± ​50.5	87.0 ​± ​64.8	0.20

*Note.* Data are shown as mean ​± ​SD; significance was tested by ANOVA.

Abbreviations: ANOVA, analysis of variance; APMAD, anteroposterior mitral annular diameter; L-MAD, large mitral annular diameter; M-TEER, mitral transcatheter edge-to-edge repair; M-MAD, medium mitral annular diameter; S-MAD, small mitral annular diameter.

**Table 3:** M-TEER outcomes by baseline APMAD

Event	30 d				2 y			
	S-MAD (N = 99)	M-MAD (N = 85)	L-MAD (N = 100)	*p* value	S-MAD (N = 99)	M-MAD (N = 85)	L-MAD (N = 100)	*p* value
Device-specific events								
SLDA	1.0% (1)	1.2% (1)	0% (0)	0.58	1.0% (1)	1.2% (1)	0% (0)	0.58
Device embolization	0% (0)	1.2% (1)	0% (0)	0.32	0% (0)	1.2% (1)	0% (0)	0.32
Any device-related complication requiring nonelective CV surgery	0% (0)	1.2% (1)	0% (0)	0.31	0% (0)	1.2% (1)	0% (0)	0.31
Progressive HF unrelated to device complications	0% (0)	0% (0)	0% (0)	-	3.9% (3)	2.7% (2)	5.2% (4)	0.81
LVAD	0% (0)	0% (0)	0% (0)	-	2.6% (2)	2.7% (2)	2.8% (2)	0.97
Heart transplantation	0% (0)	0% (0)	0% (0)	-	1.3% (1)	0% (0)	2.4% (2)	0.42

*Note.* Data are shown as % (n); significance was tested by chi-square or Fisher exact test (as appropriate).

Abbreviations: APMAD, anteroposterior mitral annular diameter; CV, cardiovascular; HF, heart failure; L-MAD, large mitral annular diameter; LVAD, left ventricular assist device; M-MAD, medium mitral annular diameter; M-TEER, mitral transcatheter edge-to-edge repair; S-MAD, small mitral annular diameter; SLDA, single leaflet device attachment.

### All-Cause Mortality and Heart Failure Hospitalizations

**Figure 1:**
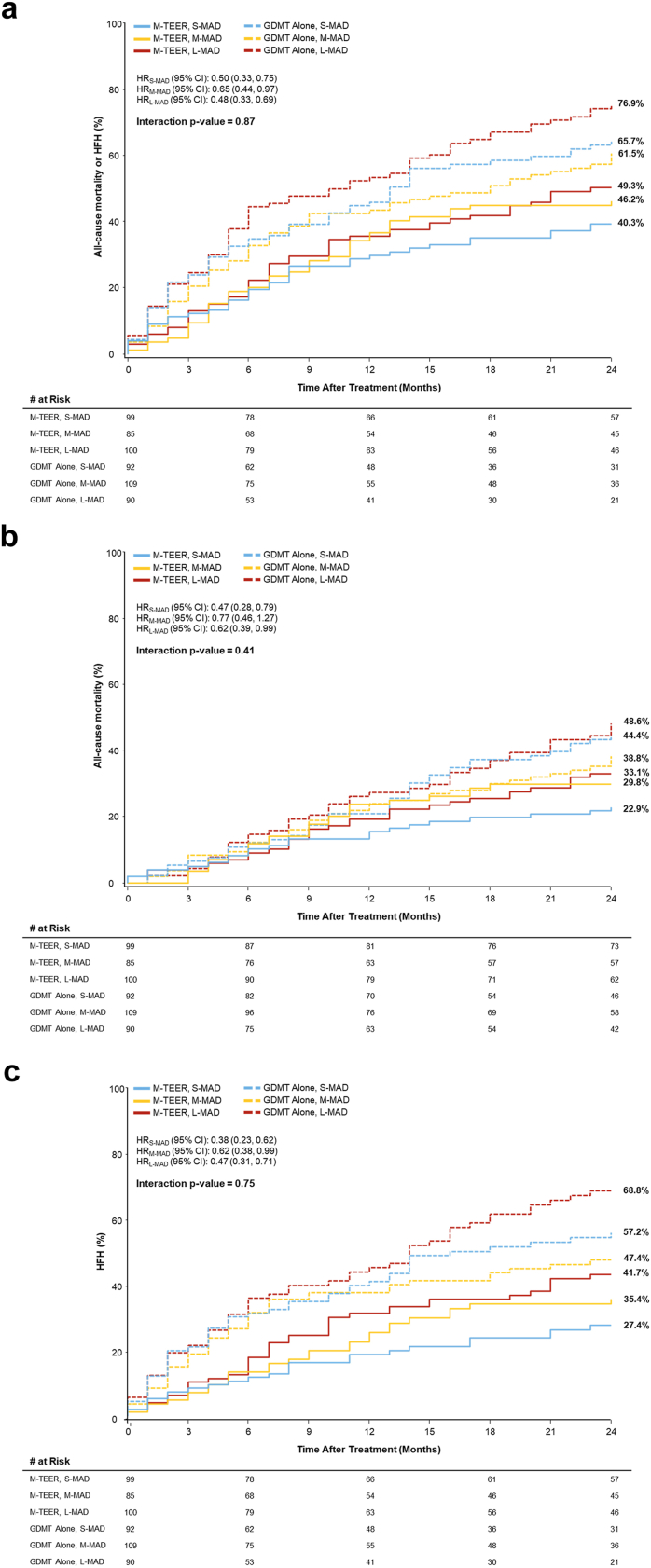
All-cause mortality and heart failure hospitalization through 2 years by APMAD and treatment. (a) Composite all-cause mortality or HFH. (b) All-cause mortality. (c) HFH. Abbreviations: APMAD, anteroposterior mitral annular diameter; GDMT, guideline-directed medical therapy; HFH, heart failure hospitalization; HR, hazard ratio; L-MAD, large mitral annular diameter, M-MAD, medium mitral annular diameter, M-TEER, mitral transcatheter edge-to-edge repair; S-MAD, small mitral annular diameter.

The summary of clinical outcomes is presented in Table 4. At 2 years, the composite rates of all-cause mortality or HFH in patients treated with M-TEER were not significantly different between groups (S-MAD, M-MAD, L-MAD: 40.3, 46.2, 49.3%, *p* ​= ​0.54). However, in patients treated with GDMT alone, the L-MAD group had a significantly higher incidence of the composite endpoint of death or HFH event rates than the M-MAD group (M-MAD: 61.5%, L-MAD: 76.9%, p _M-MAD_ vs._L-MAD_ ​= ​0.03). The composite rate of all-cause mortality or HFH was consistently reduced with M-TEER compared with GDMT alone regardless of baseline APMAD (p_interaction_ = 0.87) (Figure 1a). Similarly, the 2-year rates of all-cause mortality and HFH alone were consistently reduced with M-TEER compared with GDMT alone (*p*_interaction_ = 0.41 and *p*_interaction_ = 0.75, respectively) (Figure 1b and c). The results remained consistent after adjusting for baseline characteristics across the 3 groups. Patients treated with MitraClip had similar composite all-cause mortality or HFH event rates regardless of baseline APMAD, while patients treated with GDMT alone exhibited the highest event rates, particularly among those with the largest baseline APMAD (Supplemental Figure 2).

**Table 4:** Clinical outcomes by baseline APMAD

	S-MAD			M-MAD			L-MAD			*p*_interaction_
	M-TEER plus GDMT (N = 99)	GDMT alone (N = 92)	HR (95% CI)	M-TEER plus GDMT (N = 85)	GDMT alone (N = 109)	HR (95% CI)	M-TEER plus GDMT (N = 100)	GDMT alone (N = 90)	HR (95% CI)	
All-cause mortality or HFH	40.3% (39)	65.7% (59)	0.50 (0.33, 0.75)	46.2% (39)	61.5% (63)	0.65 (0.44, 0.97)	49.3% (48)	76.9% (68)	0.48 (0.33, 0.69)	0.87
All-cause mortality	22.9% (22)	44.4% (39)	0.47 (0.28, 0.79)	29.8% (25)	38.8% (39)	0.77 (0.46, 1.27)	33.1% (32)	48.6% (41)	0.62 (0.39, 0.99)	0.41
All-cause hospitalization	62.9% (59)	77.5% (67)	0.70 (0.49, 0.99)	67.5% (56)	77.8% (78)	0.79 (0.56, 1.11)	75.5% (73)	86.5% (74)	0.79 (0.57, 1.09)	0.69
HF-related hospitalization	27.4% (24)	57.2% (48)	0.38 (0.23, 0.62)	35.4% (27)	47.4% (47)	0.62 (0.38, 0.99)	41.7% (38)	68.8% (55)	0.47 (0.31, 0.71)	0.75

*Note.* Data are shown as % (n); significance was tested by chi-square or Fisher exact test (as appropriate).

Abbreviations: APMAD, anteroposterior mitral annular diameter; GDMT, guideline-directed medical therapy; HF, heart failure; HFH, heart failure hospitalization; L-MAD, large mitral annular diameter; M-MAD, medium mitral annular diameter; M-TEER, mitral transcatheter edge-to-edge repair; S-MAD, small mitral annular diameter.

### Echocardiographic Outcomes

**Figure 2:**
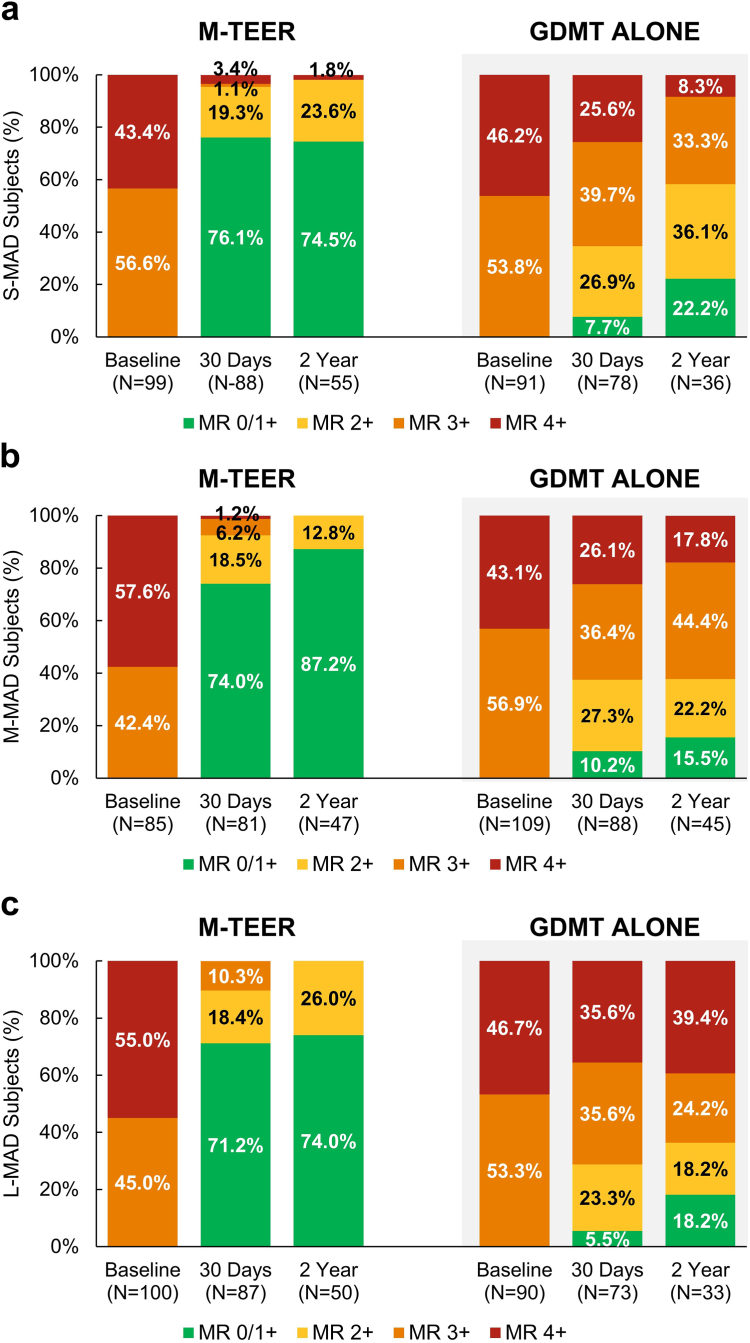
Mitral regurgitation (MR) severity by APMAD and treatment. Baseline, 30-day, and 2-year MR severity in patients with S-MAD (a), M-MAD (b), and L-MAD (c). Abbreviations: APMAD, anteroposterior mitral annular diameter; GDMT, guideline-directed medical therapy; L-MAD, large mitral annular diameter; M-MAD, medium mitral annular diameter; M-TEER, mitral transcatheter edge-to-edge repair; S-MAD, small mitral annular diameter.

At 2 years, the success rates for achieving MR ​≤ 2+ were significantly higher with M-TEER compared to GDMT alone regardless of annular dimensions: 98.2 vs. 58.3% for S-MAD, 100.0 vs. 37.8% for M-MAD, and 100.0 vs. 36.4% for L-MAD (all *p* ​< ​ 0.0001) (Figure 2a-c). Patients treated with M-TEER in the L-MAD group experienced a significant reduction in LV diastolic dimensions from baseline to 2 years (*p* = 0.003). There were no significant LV reverse remodeling effects in other groups or in the LV systolic dimensions (Supplemental Figure 3).

### Functional and Quality-of-Life Outcomes

**Figure 3:**
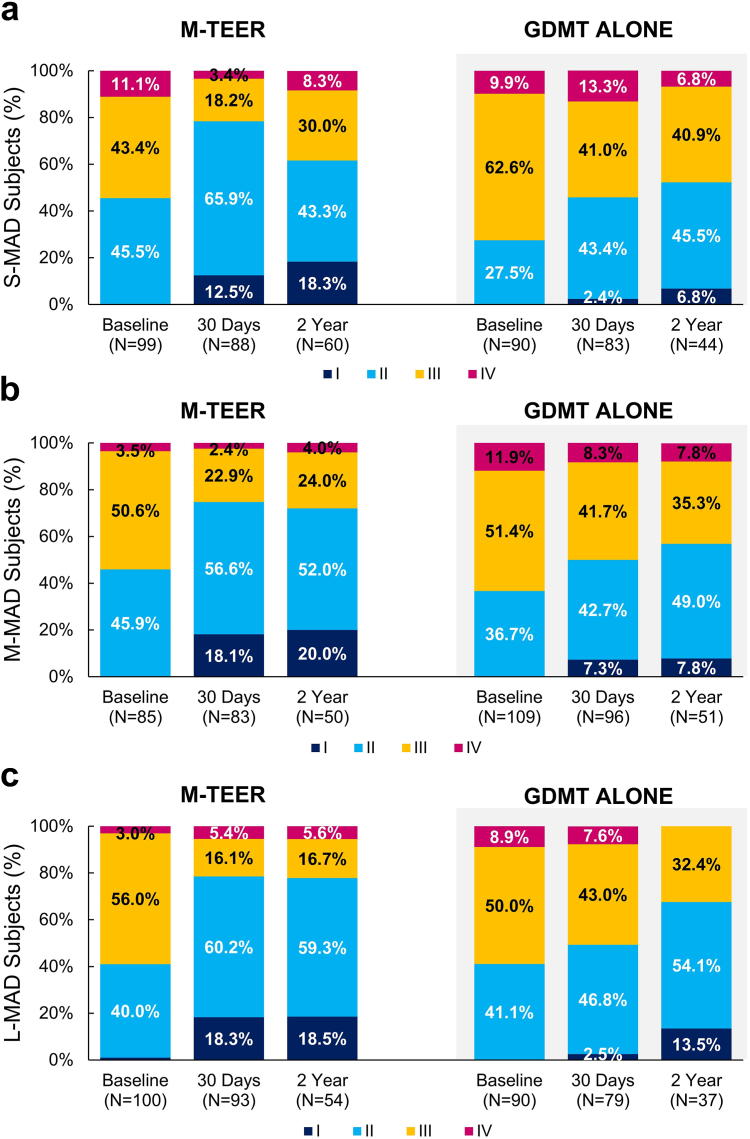
NYHA functional class severity by APMAD and treatment. Baseline, 30-day, and 2-year NYHA functional class in patients with S-MAD (a), M-MAD (b), and L-MAD (c). Abbreviations: APMAD, anteroposterior mitral annular diameter; GDMT, guideline-directed medical therapy; L-MAD, large mitral annular diameter; M-MAD, medium mitral annular diameter; M-TEER, mitral transcatheter edge-to-edge repair; NYHA, New York Heart Association; S-MAD, small mitral annular diameter.

**Figure 4:**
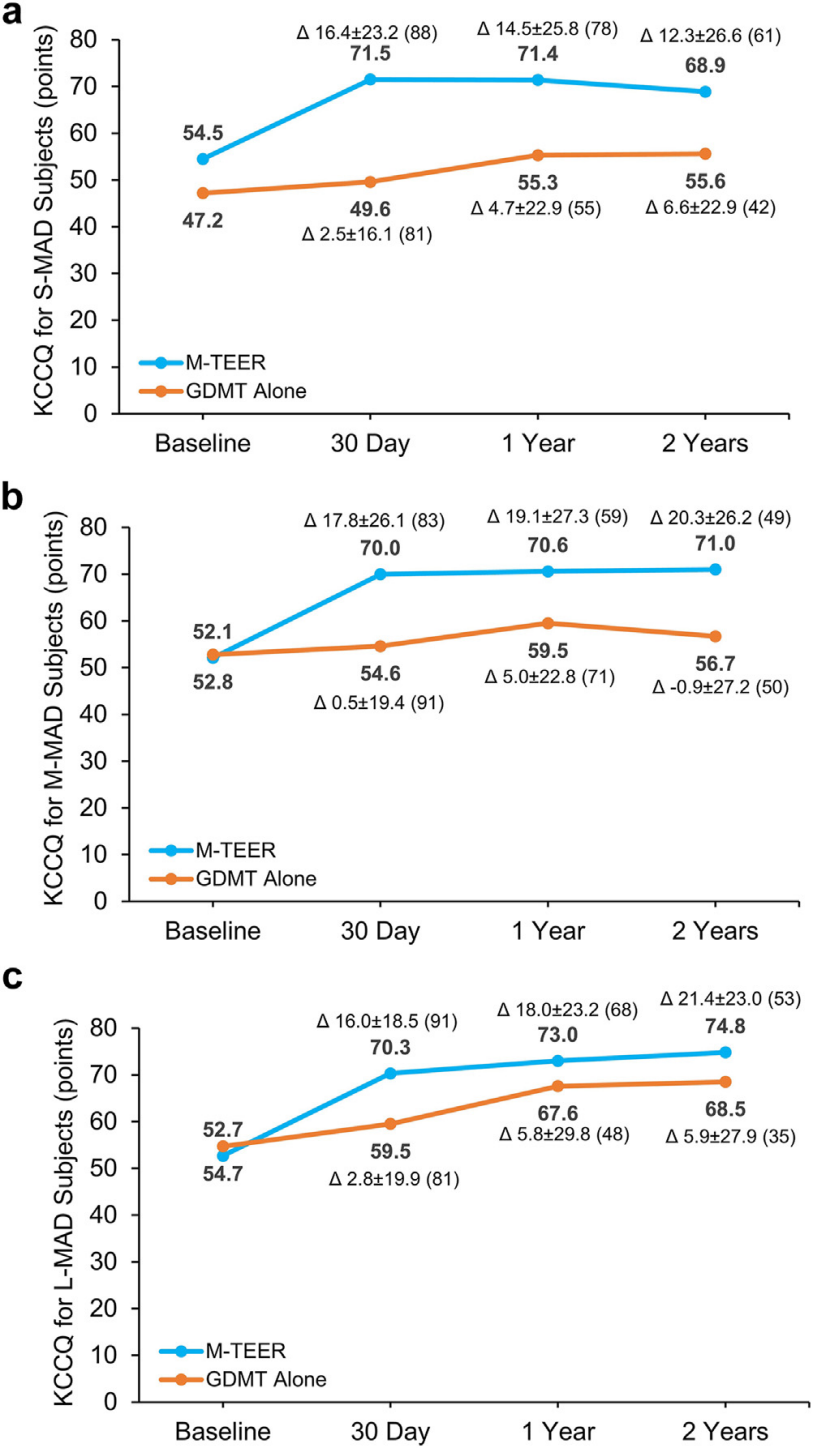
KCCQ score by APMAD and treatment. Baseline, 30-day, and 2-year KCCQ overall summary score in patients with S-MAD (a), M-MAD (b), and L-MAD (c). Abbreviations: APMAD, anteroposterior mitral annular diameter; GDMT, guideline-directed medical therapy; KCCQ, Kansas City Cardiomyopathy Questionnaire; L-MAD, large mitral annular diameter; M-MAD, medium mitral annular diameter; M-TEER, mitral transcatheter edge-to-edge repair; S-MAD, small mitral annular diameter.

At 2 years, the NYHA functional class in patients treated with M-TEER was improved from baseline regardless of baseline APMAD (Figure 3a-c). Patients treated with M-TEER experienced a large KCCQ-OS improvement from baseline to 2 years, especially in patients with larger baseline APMAD (M-MAD and L-MAD) (Figure 4a-c). The improvement in KCCQ-OS scores from baseline to 2 years was greater in patients treated with M-TEER vs. those treated with GDMT alone regardless of baseline APMAD (change in KCCQ-OS score from baseline to 2 years: S-MAD vs. GDMT: 12.3 ​± ​26.6 vs. 6.6 ​± ​22.9, *p* ​= ​0.03; M-MAD vs. GDMT: 20.3 ​± ​26.2 vs. −0.9 ​± ​27.2, *p* ​= ​0.0004; L-MAD vs. GDMT: 21.4 ​± ​23.0 vs. 5.9 ​± ​27.9, *p* ​= ​0.04) with consistent improvements across groups (*p*_interaction_ = 0.42).

## Discussion

In a previous analysis of 29 baseline transthoracic echocardiographic mitral valve parameters from the COAPT trial, larger baseline APMAD was the only independent echocardiographic predictor of the composite endpoint of all-cause mortality or HFH at 2 years.^20^ In the present analysis, patients treated with GDMT alone with the largest APMADs had the highest incidence of the composite endpoint of death or HFH. M-TEER consistently reduced MR severity and the 2-year risk of death or HFH, regardless of baseline APMAD.

SMR occurs in the setting of LV dysfunction and dilation, which in turn leads to mitral annular dilation, papillary muscle displacement, leaflet tethering, and impaired leaflet coaptation.^22^ Mitral annular dilation is related to the degree of LV enlargement, left atrial enlargement, presence of atrial fibrillation, and chronic LV volume overload. Surgical mitral annuloplasty can normalize mitral annular dimensions and restore leaflet coaptation in properly selected patients.^23^ Numerous reports utilizing modern ring surgical mitral annuloplasty have established the efficacy of annular reduction in reducing SMR; however, annuloplasty is less effective in select anatomic subsets, such as increased tenting area, higher posterior leaflet angle, and increased annular diameter.^9^ Mechanistically, the persistence of MR postsurgical annuloplasty is most frequently due to continued leaflet tethering with failure to restore the zone of coaptation.^24^ Moreover, MR can recur if there is progressive LV remodeling and enlargement with increasing leaflet tethering in the setting of chronic HF.^25^

The mechanism of M-TEER is distinctly different than annuloplasty in that the TEER device directly grasps both leaflets, pulling them together and restoring leaflet coaptation at the zone of regurgitation. In our study, we investigated the influence of baseline APMAD on M-TEER outcomes in patients with SMR compared with GDMT alone. The average APMAD is 26-30 mm in healthy patients and is larger in men compared to women.^26^ The COAPT trial did not define specific mitral valve anatomic exclusion criteria (aside from requiring the absence of primary valve disease) but did exclude leaflet anatomy that may preclude MitraClip implantation, proper positioning, or sufficient reduction in MR per site and operator assessment. Based on these assessments and other study parameters, a wide range of APMADs were enrolled in the COAPT trial; however, screened patients with the largest baseline APMADs (e.g., ​> ​40 mm) may not have been included as the leaflets may not have been assessed as graspable.

In this analysis, at 2 years, there were no significant differences in MR severity among patients treated with GDMT alone, but the composite endpoint of death or HFH was higher in patients with the largest baseline APMAD. Conversely, there were no significant differences among tertiles in MR severity and the composite endpoint of death or HFH in the device group. This suggests that patients with the largest ventricles and most severe MR still derive substantial benefit from M-TEER over GDMT alone. Nonetheless, we found a consistent benefit of M-TEER in improving freedom from death, HFH, and the composite death or HFH at 2 years in all three APMAD tertiles compared with GDMT alone. A larger study population may have been necessary to elicit whether patients with larger APMADs benefit to a greater degree with M-TEER than those with smaller APMADs.

With regard to recommendations for clinicians based on these data, the baseline measurement of APMAD can be of clinical utility, with demonstrated effectiveness of M-TEER across the wide range of diameters presented herein. For diameters larger than 40 mm, clinical judgment is advised since the COAPT trial did not enroll patients with extremely large baseline APMADs. In addition to GDMT, whether these patients will derive similar clinical benefits with M-TEER, or should instead be considered for other transcatheter mitral therapies versus advanced HF therapies, will be a subject for future investigations.

### Limitations

The COAPT trial included the first-generation MitraClip device with a single clip size (NT), and outcomes herein may not reflect those observed with the current fourth-generation G4 system which has 4 clip sizes (NT, NTW, XT, and XTW). Longitudinal change in APMAD was not assessed over time, although prior reports have suggested that APMAD decreases after M-TEER with a greater reduction with the G4 system (as compared with the first-generation MitraClip system).^19^ The COAPT trial did not include patients with very large APMADs (e.g., ≥40 mm); these patients may respond differently to M-TEER plus GDMT vs. GDMT alone. However, in this study, we did not identify an APMAD above which M-TEER was ineffective. Whether or not there is an APMAD above which efficacy with M-TEER is minimized should be examined in future investigations. Investigators and the echocardiographic core laboratory were not blinded to treatment; however, a central events committee and independent echocardiographic core laboratory were used to minimize bias. Finally, the analyses herein were performed post hoc; the original COAPT trial was not powered for this subanalysis, and results should be considered as hypothesis-generating.

### Conclusions

Mitral annular dilation in patients with SMR is associated with more advanced LV dilation, mitral leaflet tethering, and increased severity of MR. The COAPT trial enrolled patients with a wide spectrum of baseline APMADs that were treated successfully with M-TEER. Patients with the largest baseline APMADs had worse clinical outcomes with GDMT alone. However, regardless of the baseline APMAD, patients treated with M-TEER with the first-generation MitraClip system experienced substantial and consistent improvements in MR severity and the composite risk of death or HFH postprocedure compared with those treated with GDMT alone.

## Review Statement

Full responsibility for the editorial process for this article was delegated to Richard Cheng, MD.

## Ethical Statement

This study was approved by the applicable local or central ethics committees and competent authorities per national requirements and complies with the latest good clinical practice standards in the Declaration of Helsinki. All subjects provided written informed consent.

## Funding

The COAPT trial was sponsored by 10.13039/100000046Abbott (Santa Clara, CA) and designed collaboratively by the principal investigators and the sponsor.

## Disclosure Statement

J.H. Rogers is a consultant for Abbott Structural Heart, Biosense Webster, and Boston Scientific. T.W. Smith is a consultant for Abbott, Gore, Johnson & Johnson, and Novo Nordisk. J.J. Bax receives speaker fees from Edwards Lifesciences and Abbott; the Department of Cardiology at Leiden University Medical Center, the Netherlands, has received unrestricted research grants from Edwards Lifesciences and Abbott. F.M. Asch’s work as director of an academic core laboratory is paid by institutional research grants (MedStar Health) from Abbott, Boston Scientific, Medtronic, Edwards Lifesciences, Neovasc, Ancora Heart, Livanova, MVRx, InnovHeart, Polares Medical, and Aria CV. D.S. Lim’s institution receives research grant on his behalf from Abbott, Corvia, Edwards, Medtronic, and V-Wave, and he receives personal consulting fees from Philips, Valgen, and Venus. N. Wong has received honoraria fees from Abbott Vascular, Boston Scientific, OrbusNeich, and Medtronic. J. Aiyer is an employee of Abbott. W.T. Abraham has received personal fees from Abbott, Edwards Lifesciences, Cordio, CVRx, Impulse Dynamics, Sensible Medical, V-Wave, and Zoll Respicardia and equity from V-Wave. J. Lindenfeld has served as a consultant for Abbott, Alleviant, AstraZeneca, Axon, Boston Scientific, Cordio, Edwards Lifesciences, Merck, Medtronic, Orchestra Biomed, V-Wave, Vascular Dynamics, and Whiteswell and received grants from AstraZeneca and Volumetrix. M.J. Mack served as coprimary investigator for the PARTNER Trial for Edwards Lifesciences and COAPT trial for Abbott and served as study chair for the Apollo trial for Medtronic. G.W. Stone has received speaker honoraria from Medtronic, Pulnovo, Abiomed, Amgen, and Boehringer Ingelheim; has served as a consultant for Abbott, Daiichi Sankyo, Ablative Solutions, CorFlow, Cardiomech, Robocath, Miracor, Vectorious, Apollo Therapeutics, Elucid Bio, Cardiac Success, Valfix, TherOx, HeartFlow, Neovasc, Ancora, Occlutech, Impulse Dynamics, Adona Medical, Millennia Biopharma, Oxitope, HighLife, Elixir, Remote Cardiac Enablement, and Aria; and has equity/options from Cardiac Success, Ancora, Cagent, Applied Therapeutics, Biostar family of funds, SpectraWave, Orchestra Biomed, Aria, Valfix, and Xenter. G.W. Stone’s employer, Mount Sinai Hospital, receives research grants from Shockwave, Abbott, Abiomed, Bioventrix, Cardiovascular Systems Inc, Philips, Biosense Webster, Vascular Dynamics, Pulnovo, and V-Wave. S.F. Bolling is a consultant for Abbott Laboratories, Edwards Lifesciences, Medtronic, AtriCure, and Gore Medical.

## Figures and Tables

**Supplementary Figure:**