# Supplementary material for: Impact of Baseline Anteroposterior Mitral Annular Dimensions on Clinical Outcomes after MitraClip for Secondary Mitral Regurgitation
Source: Struct Heart. 2025 Mar 20;9(6):100460. doi: 10.1016/j.shj.2025.100460 (PMC12207238; doi:10.1016/j.shj.2025.100460)
Supplement: Supplementary Material [file mmc1.docx]

**SUPPLEMENTAL MATERIALS**

**Impact of Baseline Antero-Posterior Mitral Annular Dimensions on Clinical Outcomes in the COAPT Trial**

Supplemental Figure 1. Examples of mitral valve geometry measurements on two-dimensional transthoracic echocardiography…………………………………………………………………...2

Supplemental Figure 2. All-cause mortality and heart failure hospitalization through 2 years by APMAD and treatment arm and adjusted for differences in baseline characteristics…………….3

Supplemental Figure 3. Change in indexed left ventricular dimensions from baseline to 2 years by APMAD and treatment arm……………………………………………………………………4

**
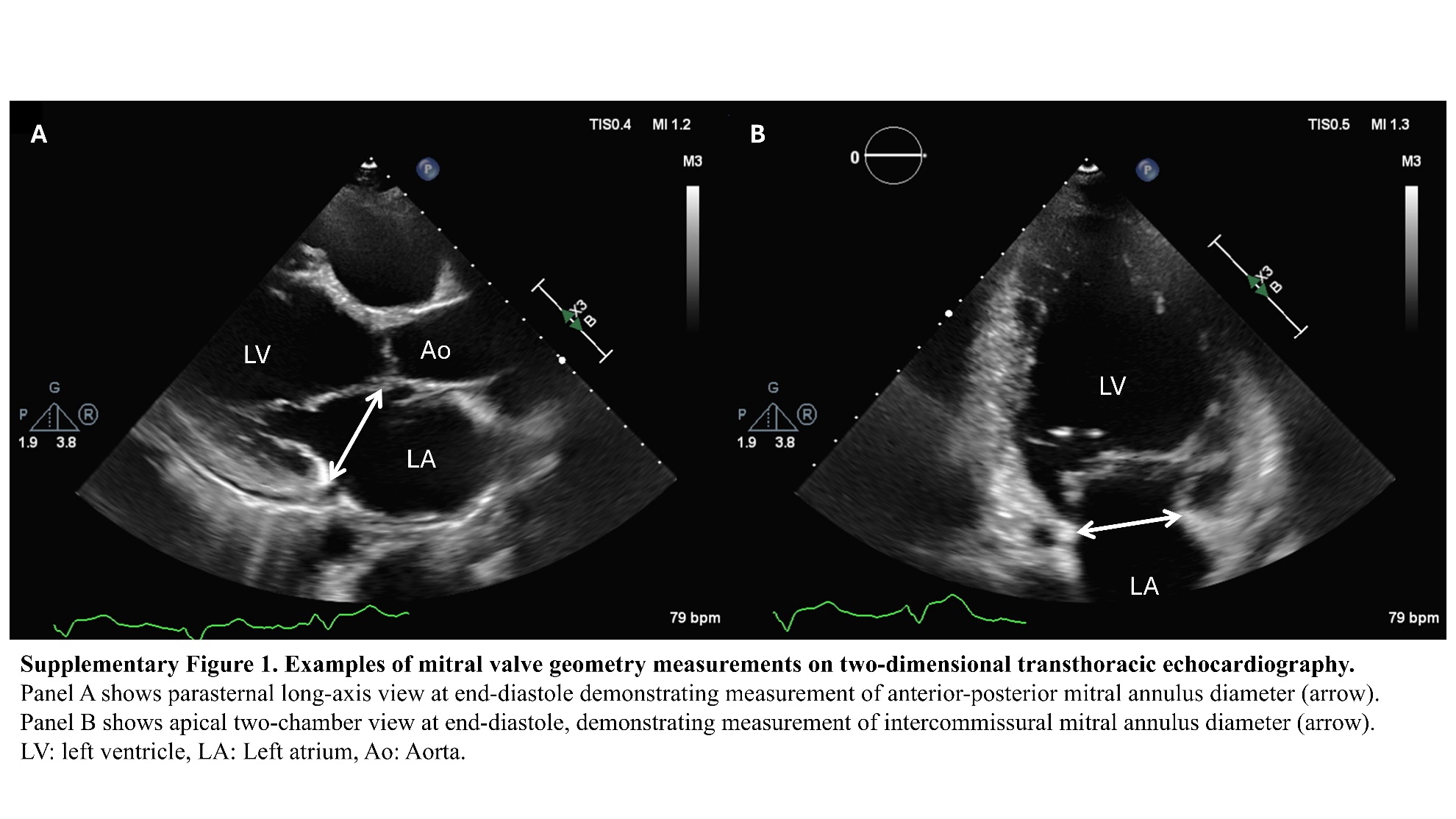
 Supplementary Figure 1. Examples of mitral valve geometry measurements on two-dimensional transthoracic echocardiography.** Panel A shows parasternal long-axis view at end-diastole demonstrating measurement of anterior-posterior mitral annulus diameter (arrow). Panel B shows apical two-chamber view at end-diastole, demonstrating measurement of intercommissural mitral annulus diameter (arrow). LV: left ventricle, LA: Left atrium, Ao: Aorta.


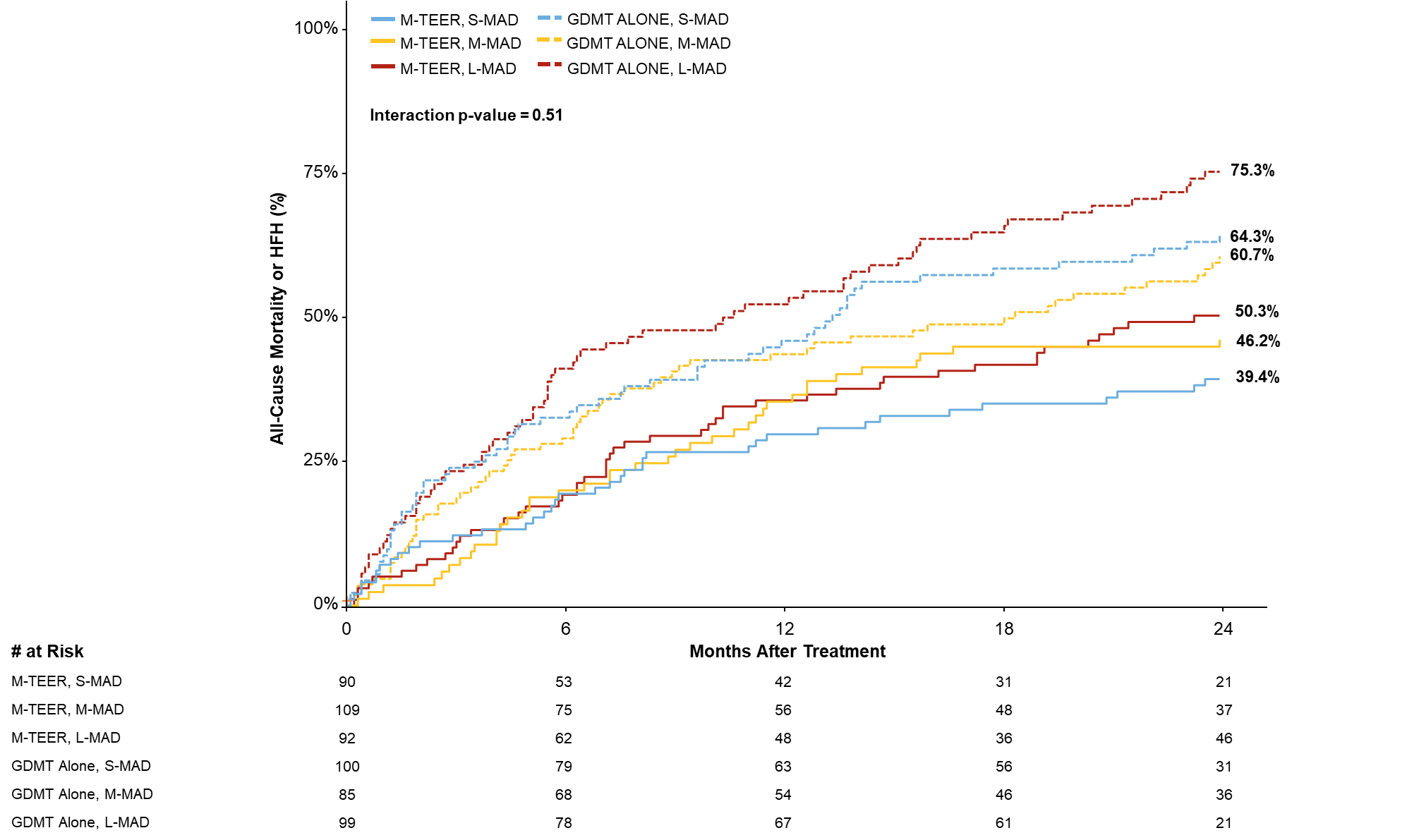


**Supplementary Figure 2. All-cause mortality and heart failure hospitalization through 2 years by APMAD and treatment arm and adjusted for differences in baseline characteristics.** The analysis was adjusted for the following characteristics: sex, diabetes, renal disease, and atrial fibrillation.

**
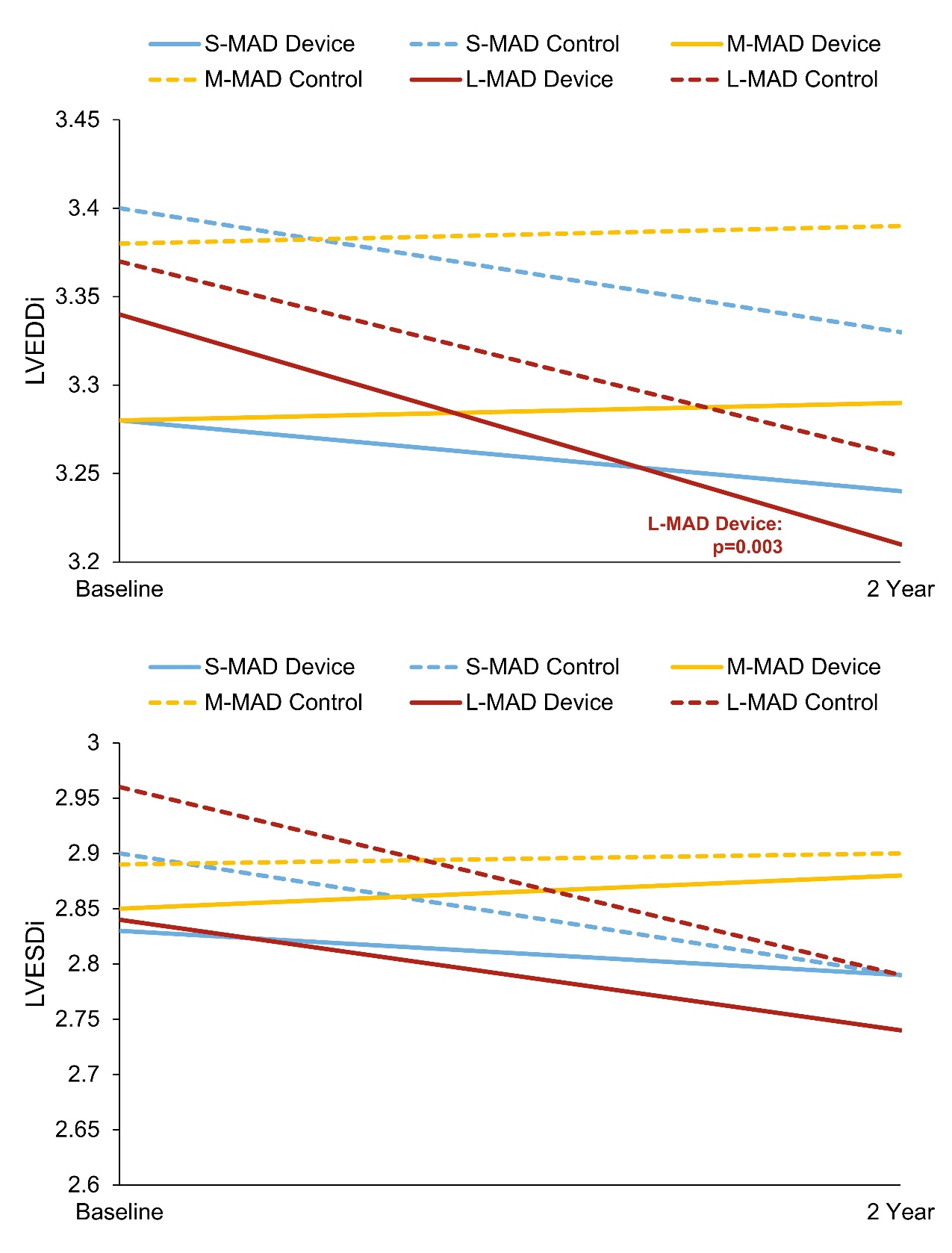
Supplementary Figure 3. Change in indexed left ventricular dimensions from baseline to 2 years by APMAD and treatment arm.** Indexed left ventricular end diastolic dimension (LVEDDi) was significantly different at 2 years for the L-MAD Device group; significant differences were not observed for other groups for LVEDDi through 2 years, nor observed for any groups for indexed left ventricular end systolic dimension (LVESDi).
